# The incidence of vascular injuries in patients with negative cervical computed tomography (CT) following blunt trauma

**DOI:** 10.1007/s10140-024-02310-5

**Published:** 2025-01-14

**Authors:** Assala Aslan, Joseph Eskew, Spencer Zaheri, Ridge Arceneaux, Elizabeth Field, Elise Thibodeaux, Morgan Roque, Luis De Alba, Octavio Arevalo, Hugo Cuellar

**Affiliations:** Louisiana State University Health Sciences Center , Shreveport, USA

**Keywords:** Blunt, Trauma, Cervical, Vascular, Vascular

## Abstract

**Introduction:**

Computed tomography (CT) angiography is commonly utilized to quickly identify vascular injuries caused by blunt cervical trauma. It is often conducted alongside a cervical spine CT, based on established criteria. This study assessed the prevalence of cervical vascular injuries identified via CT angiography (CTA) in patients who had negative findings on cervical CT scans.

**Materials and methods:**

A retrospective study was performed on patients who experienced blunt trauma from January 2020 to December 2022 and underwent both cervical CT and CTA. The sample size was determined using the formula: n = (Z^2 * P * (1 - P)) / E^2, assuming a 99% confidence interval, a 2% margin of error, and a proportion of 0.05.

**Results:**

A total of 1,165 patients presented with acute blunt trauma to the head and neck during the study period. Out of those, 800 patients (68.7%) had negative cervical CT scans and only 5 patients (0.6%) were found to have vascular injuries on CTA, with an average age of 44.2 years. Regarding the severity of the injuries, three were classified as grade I and two as grade II. On the other hand, of the 365 patients with positive cervical CT, 44 patients (12%) had vascular injury on CTA, including 16 patients (4.5%) with grades III and IV injuries.

**Conclusion:**

The findings of this study suggest that CTA in patients with negative cervical CT scans seldom reveals vascular injuries, with no injuries exceeding grade II. This highlights the selective utility of CTA in this patient group.

## Introduction

Blunt trauma to the cervical spine predominantly occurs in scenarios such as high-speed motor vehicle collisions or significant falls. These events can lead to vascular injuries in the vertebral and carotid arteries. When associated with cervical spine fractures, the incidence of such vascular injuries can be as high as 30%, occasionally constituting a life-threatening emergency. The urgent detection and treatment of these injuries are crucial to prevent the onset of severe ischemic complications that can result in long-term morbidity or even mortality [1].

Historically, the Denver criteria set forth guidelines mandating CT angiography (CTA) screening for patients presenting with high-risk injuries, including C1–C3 fractures, fractures impacting the foramen transversarium, and cervical fracture subluxations [2]. Over time, these criteria were expanded to include non-fracture indications, thus widening the scope of screening to potentially capture more cases of vascular injury [3]. Despite the broader application of these guidelines, there remains a notable lack of empirical evidence supporting their use, especially among patients who show no apparent abnormalities such as fractures, dislocation, or malalignment on conventional cervical CT scans. This gap in evidence raises questions about the necessity and efficiency of widespread CTA in contexts where initial imaging results are unremarkable [4].

In this study, we aim to investigate the incidence and nature of vascular injuries in patients who have sustained blunt cervical trauma but present with negative findings on cervical CT scans. Our goal is to ascertain whether the expanded use of CTA is justified in such cases and to refine screening protocols to ensure they are both clinically relevant and cost-effective. This research not only seeks to validate current medical practices but also to potentially recalibrate the criteria used for deploying advanced imaging techniques in the assessment of cervical spine trauma.

## Methods

At our our level 1 trauma center, trauma patients are classified into three tiers: Tier I: Life-threatening injuries with unstable vital signs, Tier II: Potentially life-threatening injuries with stable vital signs, and Tier III: No obvious life-threatening injuries with stable vital sign. All patients with direct trauma to the neck get concurrent CT and CTA of the cervical spine. In this study, we conducted a retrospective analysis of a prospectively maintained database, to identify patients with cervical blunt trauma who sustained cervical vascular injury in the absence of cervical fracture. All blunt trauma patients above the age of 18 years, who presented to our center as tiers 1, 2, and 3 injuries between January 2020 and December 2022 were reviewed. Inclusion criteria included concurrent CT cervical spine and CTA. Patients were excluded from the study if they did not have both a cervical CT and CTA available, if the imaging was not performed concurrently, or if the patient had prior history of cervical spine fusion.

The data collected included patient characteristics (e.g. age, gender), trauma details (e.g. mechanism, tier), and imaging details (CT and CTA findings). The Biffle scale was used to assess the degree of vascular injury [5]. All images were originally reviwed and reported by specializaed neuroradiologists. If the report indicated vascular inuury, it was re-reviewed by the senior author to confirm the findings. The sample size required for this study was calculated using the formula: n = (Z^2 * P * (1 - P)) / E^2. Assuming a 99% confidence interval, we used the following values: Z (Z-score for a 99% confidence interval) = 2.326, P (proportion) = 0.05, and E (margin of error) = 2%. Based on these parameters, the calculated sample size (N) was 791.

The study hypothesis was that in patients with a negative cervical CT, the incidence of detecting a vascular injury of grade II or higher using CTA would be less than 5%. All procedures and data handling methodologies were reviewed and approved by the Institutional Review Board (IRB) before the commencement of the study, ensuring compliance with ethical standards and patient confidentiality.

## Results

**Fig. 1:**
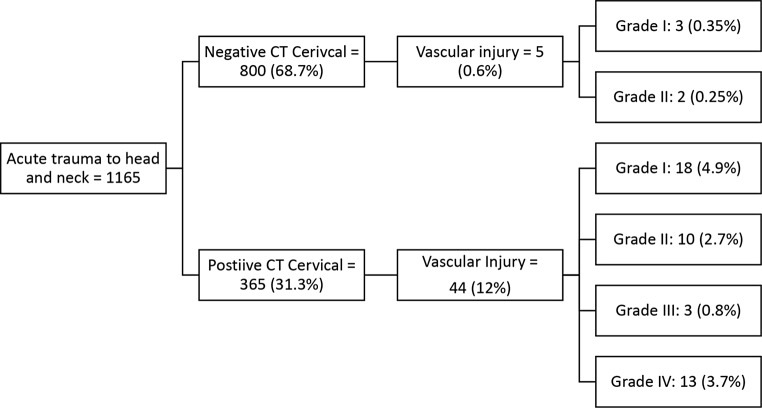
A diagram illustrating the cases inclusion and breakdown

During the period from January 2020 to December 2022, a substantial cohort of 9,632 patients were admitted to our hospital with acute trauma. Among these, 1,165 patients (12.1%) sustained blunt trauma specifically to the head and neck and consequently underwent both cervical CT and CTA as part of their diagnostic evaluation. Of these 1,165 patients, 800 patients (68.7%) were found to have negative results on their cervical CT scans, indicating no evidence of fractures, dislocations, or malalignment (Fig. 1).

**Fig. 2:**
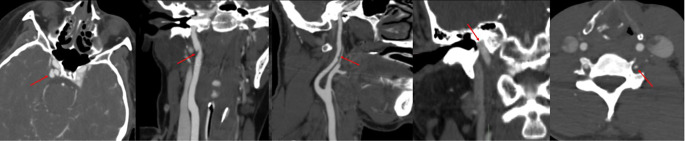
The five cases of vascular injury in the absence of craniocervical fracture

Within this subgroup of 800 patients with negative cervical CT results, only a small fraction, precisely 5 patients (0.6%), were subsequently found to have vascular injuries on their cervical CTA scans. This cohort comprised 2 females and 3 males, with an average age of 44.2 years, reflecting a mixed demographic. The severity of the vascular injuries identified was relatively low, with 3 of these patients presenting with grade I injuries and 2 patients with grade II injuries. Four of the injuries occurred in the internal carotid artery and 1 in the vertebral artery (Fig. 2). Further breaking down the trauma severity at the time of presentation among these 5 patients, 3 were classified under tier 1 trauma, indicating the most severe trauma classification in our protocol, while the remaining 2 were classified as tier 2.

On the other hand, of the 365 patients (31.3%) with positive CT cervical spine, 44 patients (12%) had vacular injury on CTA: 18 (4.9%) were grade I, 10 (2.7%) grade II, 3 (0.8%) grade III, and 13 (3.7%) grade IV.

## Discussion

The current study offers significant insights into the incidence and severity of cervical vascular injuries following blunt cervical trauma in patients who show no fractures on cervical CT scans. Only 0.6% of those patients sustained vascular injury visible on CTA, and no recorded injuries above grade II. This is in contrast to a 12% risk of vascular injury in patients with positive cervical CT scans. Our findings suggest that routine CTA might not be necessary in patients who lack visible signs of cervical fractures on initial imaging, and should be considered more selectively, based on specific clinical presentations rather than as a standard response.

The potential for cervical vascular injuries following blunt trauma is notably high, with general risks estimated up to 11%, and increasing to as much as 30% when a cervical fracture is present [6, 7]. The incidence in our cohort was 12%. Such injuries are critically important to identify promptly as they can lead to ischemic strokes. These strokes often remain clinically undetected, especially in scenarios where patients are intubated and undergoing multiple interventions due to polytrauma. In these cases, CTA serves as a crucial diagnostic tool to swiftly identify vascular injuries even when overt clinical symptoms are absent, enabling the initiation of preventive therapies aimed at mitigating the progression of ischemia [7]. Furthermore, specific fracture types such as those extending into the transverse foramen dramatically increase the risk of vertebral artery injuries. Likewise, vertebral dislocations, including those involving jumped facets, can cause sudden shifts around the anchored vertebral artery within the foramen, potentially leading to dissections or other forms of injury [8].

This study appears to be the first to quantify the incidence of cervical vascular injuries among patients who have sustained blunt cervical trauma yet show no fractures on CT scans. The predominant mechanism of injury in these cases likely involves excessive shearing forces experienced during extreme movements, such as the rapid flexion and extension seen in whiplash injuries. This dynamic can result in intimal damage to the vascular walls, which was supported by our findings where all affected patients exhibited only grade I or II injuries [9]. 

These findings also highlight the current practice of performing CT cervical spine imaging and CTA concurrently, a method that has become widespread due to its efficiency in trauma settings. However, this approach raises important considerations regarding radiation exposure, patient transport logistics, and overall workflow management in emergency departments. While the simultaneous use of CT and CTA offers advantages in speed and comprehensive assessment, it also results in higher cumulative radiation doses to patients. This concern is particularly relevant when the CTA may not yield significant clinical findings, as shown by our study’s results: only 0.6% of patients without fractures on initial CT scans exhibited vascular injuries, and all identified injuries were limited to grade I or II severity. These findings suggest that routine use of CTA should be more selectively applied, especially in cases without evidence of fractures.

This study has two key implications: first, it questions the routine use of CTA when CT scans do not show fractures, advocating instead for a more selective approach based on clinical judgment and individual patient factors. Second, it highlights the need to identify subtle but potentially serious vascular injuries in patients with significant blunt cervical trauma. This refined understanding can lead to more precise and effective clinical interventions, ultimately improving outcomes for this challenging group of trauma patients.

### Limitations

The study presents some limitations that must be considered when interpreting the findings. Firstly, the retrospective design of the study inherently limits our ability to control for all potential confounding variables that might influence the outcomes. Another significant limitation is the single-center nature of the study. The data was collected from only one hospital, which may limit the generalizability of the results. Hospitals vary in patient demographics, trauma severity, and clinical practices. Additionally, the interpretation of imaging studies in our research was conducted by multiple radiologists whose levels of experience varied. This variation could lead to inconsistencies in the interpretation of CT and CTA scans, potentially affecting the reliability of the data on detected injuries. Differences in diagnostic accuracy and observational skills among radiologists might result in either overestimation or underestimation of the prevalence of vascular injuries in the studied population.

## Conclusion

The findings of this study underscore the selective utility of CTA in detecting vascular injuries among patients who have experienced blunt cervical trauma but show negative results on cervical CT scans. Only a small fraction, 0.6%, of these patients had detectable vascular injuries on CTA, all of which were of low severity (not exceeding grade II). These results suggest that the routine application of CTA in all patients with blunt cervical trauma and negative initial CT scans may not be necessary or cost-effective, supporting a more targeted approach based on specific clinical indicators such as injury mechanism, symptoms, and other risk factors. This approach could optimize resource use and minimize unnecessary radiation exposure without compromising patient care. Given the critical nature of undetected vascular injuries, further research is essential to refine guidelines for using CTA, aiming to better identify those patients who would benefit most from this diagnostic tool.

## Data Availability

No datasets were generated or analysed during the current study.
